# Targeting TR4 nuclear receptor with antagonist bexarotene increases docetaxel sensitivity to better suppress the metastatic castration-resistant prostate cancer progression

**DOI:** 10.1038/s41388-019-1070-5

**Published:** 2019-11-20

**Authors:** Linyi Hu, Yin Sun, Jie Luo, Xiang He, Meihua Ye, Gonghui Li, Yong Zhang, Jian Bai, Dahong Zhang, Chawnshang Chang

**Affiliations:** 1 Department of Urology, Zhejiang Provincial People’s Hospital, Hangzhou, 310014 China; 2 George Whipple Lab for Cancer Research, Departments of Urology and Pathology and The Wilmot Cancer Institute, University of Rochester Medical Center, Rochester, NY USA; 3 Department of Urology, The Second Hospital of Hebei Medical University, Shijiazhuang, 050000 China; 4 Sex Hormone Research Center, Department of Urology, China Medical University and Hospital, Taichung, 404 Taiwan

**Keywords:** Cancer therapeutic resistance, Prostate cancer

## Abstract

Prostate cancer (PCa) is the second leading cause of cancer death in men in America, and there are no curative options for metastatic castration-resistant prostate cancer (mCRPC). Docetaxel (DTX) has been used as a standard chemotherapy for the mCRPC. However, resistance to DTX is a significant clinical problem as half of patients fail to respond to therapy. The TR4 nuclear receptor has been reported to play an important role in PCa progression, however, its linkage to the DTX resistance remains unclear. Here we found that TR4 was upregulated after DTX chemotherapy in the mCRPC cells and patients, and TR4 expression is correlated with DTX sensitivity with a higher level conferring chemo-resistance. Targeting TR4 with an antagonist bexarotene (Bex, a derivative of retinoid) suppressed the TR4 transactivation with increased DTX chemo-sensitivity. Mechanism dissection studies revealed that TR4 might alter the DTX chemo-sensitivity via modulating the TR4/lincRNA-p21/HIF-1α/VEGF-A signaling. Together, these results suggest that targeting this newly identified TR4/lincRNA-p21/HIF-1α/VEGF-A signaling with Bex, an FDA-approved drug, may increase the DTX chemo-sensitivity to better suppress the mCRPC progression.

## Introduction

Prostate cancer (PCa) is the second leading cause of cancer death in men in the United States [1]. Although many patients have been found to have localized and potentially curable disease, a large number of deaths are driven by the development of metastatic castration-resistant PCa (mCRPC) [2], and there are no curative options for mCRPC [3]. The androgen deprivation therapy (ADT) remains the standard therapy for mCRPC, yet the resistance to ADT is inevitable [4].

Since its approval by the US Food and Drug Administration (FDA) in 2004, docetaxel (DTX) chemotherapy has become another option to treat the mCRPC. However, resistance to DTX is a significant clinical problem because half of patients treated do not respond to therapy. Moreover, even though many of the patients initially respond to therapy, many may eventually develop resistance [4]. Mechanisms of resistance to DTX are not completely understood, but a significant body of the literature has emerged addressing this issue [5, 6]. Several mechanisms of DTX resistance have been proposed, including limiting intracellular drug concentrations, antagonizing the drug-stabilizing effect on microtubules, and antagonizing or circumventing the cytotoxic effect of taxanes through alternative growth pathways or apoptotic escape [2, 7].

The testicular nuclear receptor 4 (TR4; nuclear receptor subfamily 2, group C, member 2) encodes a 67-kDa protein [8]. Members of this superfamily act as ligand-activated transcription factors and function in many biological processes, such as development, cellular differentiation, and homeostasis [9]. The activated receptor/ligand complex is translocated to the nucleus where it binds to TR4-response-elements (TR4REs) of target genes. TR4 may play a role in protecting cells from oxidative stress and damage induced by ionizing radiation [10]. The engineered deficiency of the TR4 gene in mice results in growth retardation, severe spinal curvature, subfertility, premature aging, and prostatic intraepithelial neoplasia development [11]. These in vivo functions indicated that TR4 might act as a caretaker tumor suppressor to suppress the PCa initiation via promoting DNA repair and maintaining genome integrity [12]. A clinical survey of PCa samples also found that one allele of the TR4 gene is deleted in 9% of PCa patients, indicating that TR4 could be a tumor suppressor in the development of PCa [13]. However, in vitro cell movement assays and in vivo mouse studies also indicated that TR4 could promote PCa cell migration/invasion via altering the CCL2/CCR2 axis, suggesting that TR4 can also function as an enhancer to promote the PCa metastasis [14]. Yang et al. found that TR4 may function through altering the OCT4 and IL1Rα to modulate the PCa progression [13], and TR4 could also alter the PCa cells’ radio-sensitivity and might become a prognostic indicator for PCa patients receiving radiotherapy [10].

Long non-coding RNAs (lncRNAs, lincRNAs) over 200 bp in length have been implicated as being fundamental factors in numerous molecular processes, including cell differentiation, lineage specificity, neurological disorders, and cancers [15]. In human PCa, many lncRNAs (PCA3, PCGEM1, SChLAP1, PCAT-1, and PCAT-3) play roles in PCa initiation and progression [16]. A previous report showed that exosomal lincRNA-p21 expression may help to improve the diagnosis of the malignant state for patients with PCa [17]. However, the physiological and pathological roles of lincRNA-p21 in the PCa remain unclear. A clinical study found that lincRNA-p21 levels were significantly higher in patients with PCa than with BPH [18], while another study found that lincRNA-p21 was downregulated in human PCa tissues with a low lincRNA-p21 level predicting poor survival [17]. In vitro lincRNA-p21 inhibits PCa growth through induction of apoptosis in PCa cells [19].

Here, we investigated the role of TR4 in PCa chemo-sensitivity and found that TR4 might alter the DTX chemo-sensitivity via modulating the TR4/lincRNA-p21/HIF-1α/VEGF-A signaling.

## Results

### TR4 expression in PCa cell lines and clinical tissue samples

**Fig. 1:**
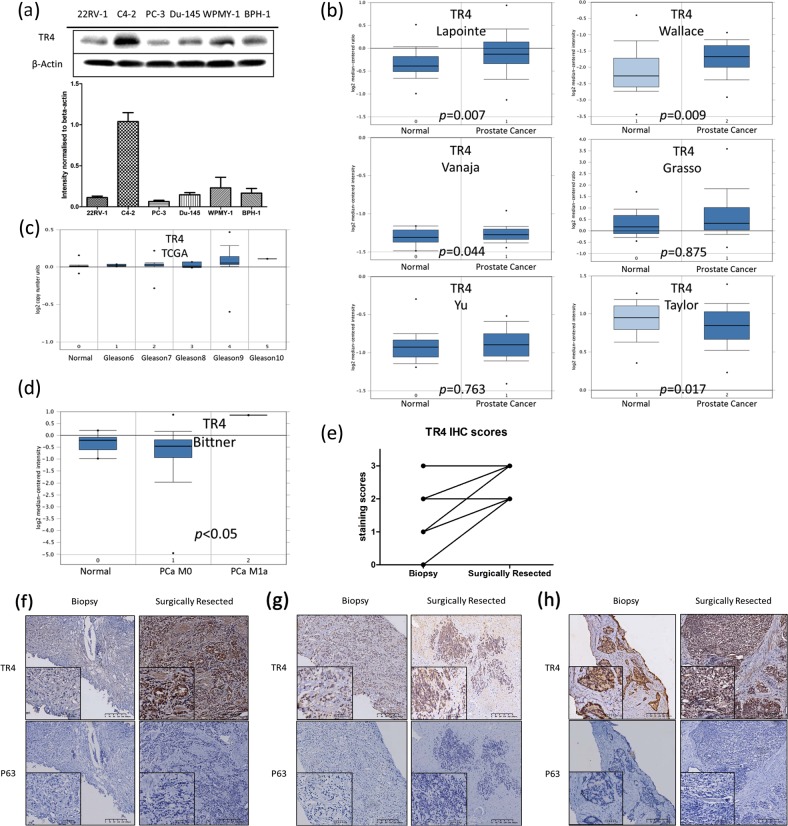
TR4 expressions in PCa cell lines and clinical tissue samples: **a** Western blot TR4 expression and quantification in four common PCa cell lines, PC-3, Du-145, C4-2, and 22RV-1, and two nontumor prostate cell lines, WPMY-1 and BPH-1. PC-3, Du-145, and 22RV-1 cells had relatively low TR4 expressions compared with C4-2 and benign prostatic cells. **b** Oncomine data mining analysis (Oncomine (https://www.oncomine.org), threshold by *P*-VALUE: 1E-4, Fold Change: 2, GENE RANK: Top 10%) of TR4 mRNA levels in six different datasets of normal tissues versus PCa samples, TR4 expression increased in PCa tissues in most of the datasets compared with normal control. **c** DNA copy number of TR4 in PCa samples from TCGA dataset showed that TR4 increased as Gleason score increased from 6 to 10. **d** Analysis of dataset from Bittner showed that metastasis PCa had higher TR4 expression than nonmetastasis ones. **e** IHC score comparison from biopsy samples with surgically resected samples in the same patients after DTX treatment from 14 PCa patients (details in Supplementary data Table S1). Quantitative scoring methods were applied as scores 0, 1+, 2+, and 3+ to represent positive cells of none or rare, <25%, 25–75%, and >75%, respectively in ×400 magnification. Paired *t*-test was used and significant difference was found, *p* < 0.001. **f**–**h** Representative images of IHC tissue staining for TR4 and P63 from three cases of patients with PCa, ×100 and ×400 magnification. TR4 antibody (ab58365, Abcam) stains nuclei, P63 (PB0164, Boster) stains nuclei and used for differentiation of PCa from benign tissues. The TR4 expressions varies in biopsy samples while high expression could be seen in DTX treated patients and most cases had TR4 upregulation. Data are presented as mean ± SD, *p* values are given in the graphs

We investigated the TR4 expression in four common PCa cell lines, PC-3, Du-145, C4-2, and CWR22RV-1 (22RV-1), as well as two nontumor prostate cell lines WPMY-1 and BPH-1. The western blot results revealed that PC-3, Du-145, and 22RV-1 cells had relatively lower TR4 expression compared with C4-2 and benign prostatic cells (Fig. 1a).

We then analyzed TR4 expression in human PCa clinical tissues from the public available database Oncomine (https://www.oncomine.org), threshold by *P*-VALUE: 1E-4, FOLD CHANGE: 2, GENE RANK: Top 10%. Results revealed that TR4 expression increased in PCa tissues as compared with normal control tissues (*p* < 0.05) (Fig. 1b). Results from TCGA analysis indicated that DNA copy number of TR4 increased as the Gleason score increased from 6 to 10 (Fig. 1c), and results from Bittner dataset analysis also revealed that metastatic PCa had higher TR4 expression than nonmetastatic tissues (Fig. 1d).

As TR4 may play roles in the PCa DTX resistance [20], we also retrospectively investigated the TR4 expression through immunohistochemical (IHC) staining of clinical samples from metastatic PCa patients treated with DTX chemotherapy. Tumor samples were collected from 14 patients, whose clinical parameters were summarized in Supplementary data Table S1. All 14 patients were first diagnosed to have high-risk PCa by fine-needle biopsy and further assays showed bone metastasis with or without liver metastasis. DTX chemotherapy was given immediately and two cases with oligo bone metastasis were given laparoscopic prostatectomy after 1 month’s chemotherapy. Other cases were given Transurethral Resection of the Prostate due to severe obstruction in urination or urinary retention. We then compared the TR4 expression from biopsy samples with surgically resected samples in the same patients after DTX treatment. The results revealed that most cases had TR4 upregulation after DTX treatment (Fig. 1e, *p* < 0.001). P63 was stained for differentiation of PCa from benign tissues. The TR4 expressions varied in biopsy samples while high expression could be seen the samples from DTX treated patients (Fig. 1f–h).

Together, the results from human clinical survey (Fig. 1a–h) suggest that TR4 may play an important role to alter the DTX chemo-sensitivity in the high-risk metastatic PCa patients.

### TR4 increases chemo-resistance in PCa cell lines

**Fig. 2:**
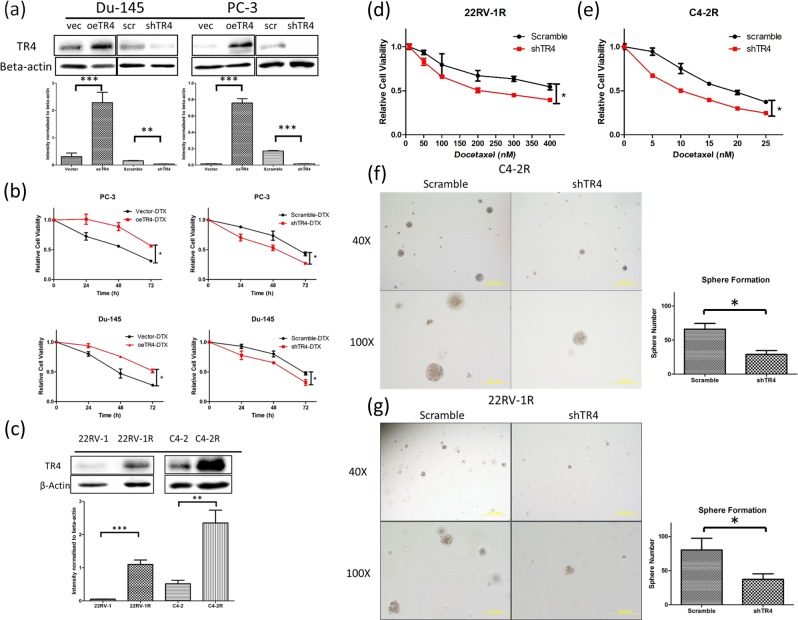
TR4 increased chemo-resistance in PC-3 and Du-145 cells and TR4 role in docetaxel-resistant C4-2, 22RV-1 cells: **a** TR4 expression and quantification of western blots of PC-3 and Du-145 cells after overexpressing TR4 (oeTR4) and silencing TR4 (shTR4) using lentivirus infection. **b** Adding oeTR4 increased chemo-resistance while shTR4 increased chemo-sensitivity in PC-3 and Du-145 cells treated with 2 nM docetaxel. **c** TR4 expression and quantification was significantly increased in the two DTX-resistant PCa cell lines as compared with their DTX-sensitive parental PCa cell lines C4-2 and 22RV-1. **d, e** Knocking down TR4 could reduce docetaxel resistance in C4-2R and 22RV-1R cells. **f, g** Tumor initiating cells (TICs) were linked to the chemo-resistance and sphere formation was used to identify the TICs. After being checked under microscope for single-cell suspension, cells were plated in ultra-low adhesion 48-well plates at a density of 1 × 10^3^ cells/well and grown in a serum-free RPMI-1640 and matrigel (BD Biosciences, 1:1) media containing 20 ng/ml of epidermal growth factor (EGF, R&D Systems, Minneapolis, MN, USA). After 7 days in culture, the number of spheres was quantified by counting spheres in three random fields (×40) under a phase contrast microscope. Knocking down TR4 could reduce sphere formation numbers in C4-2R and 22RV-1R cells, indicating targeting TR4 may lead to reducing the TICs and chemo-resistance in the DTX-resistant PCa cells. Data are presented as mean ± SD, *p < 0.05, **p < 0.01, ***p < 0.001

To confirm the human clinical study showing TR4 may increase DTX chemo-resistance in the PCa cell lines, we first transduced PC-3 and Du-145 cells with TR4 overexpressing (oeTR4) and TR4 silencing (shTR4) lentivirus or control vectors. Western blot results confirmed the efficiency of transductions showing significant increases and decreases in TR4 protein levels in PC-3 and Du-145 cells when infected with oeTR4 and shTR4 lentivirus, respectively (Fig. 2a) compared with control vectors. Then we studied the impact of altered TR4 expression on the DTX sensitivity for PCa cells, and results revealed that adding TR4 via oeTR4 increased chemo-resistance, while knocking down TR4 via shTR4 could increase chemo-sensitivity (Fig. 2b) in both cell lines. In contrast, we found little impact of TR4 on cell viability via either shTR4 or oeTR4 in the cell controls without DTX treatment.

Together, results from Fig. 2a, b suggest that TR4 may have an important role to alter DTX sensitivity for suppression of the PCa cell viability.

### Targeting TR4 led to reducing the TICs and chemo-sensitivity in the DTX-resistant PCa cells

In addition to altering the DTX chemo-sensitivity, we are interested to see the impact of targeting TR4 in DTX-resistant PCa cells. We first found that TR4 expression was significantly increased in the two DTX-resistant PCa cell lines (C4-2-R and 22RV-1-R) as compared with their parental DTX-sensitive PCa cell lines, C4-2 and 22RV-1 (Fig. 2c). Results from the cell viability assay revealed that knocking down TR4 led to reduce the DTX resistance in C4-2-R and 22RV-1-R and C4-2-R cells (Fig. 2d, e). Since tumor initiating cells (TICs) were linked to the chemo-resistance [21, 22], and sphere formation was used to identify the TICs characteristic of self-renewal in vitro, we also knocked down TR4 in PCa cells and results revealed suppressing TR4 reduced sphere formation in C4-2-R and 22RV-1-R cells (Fig. 2f, g), indicating targeting TR4 may reduce the TICs and chemo-resistance in the DTX-resistant PCa cells.

### TR4 antagonist bexarotene (Bex) increases DTX chemo-sensitivity

**Fig. 3:**
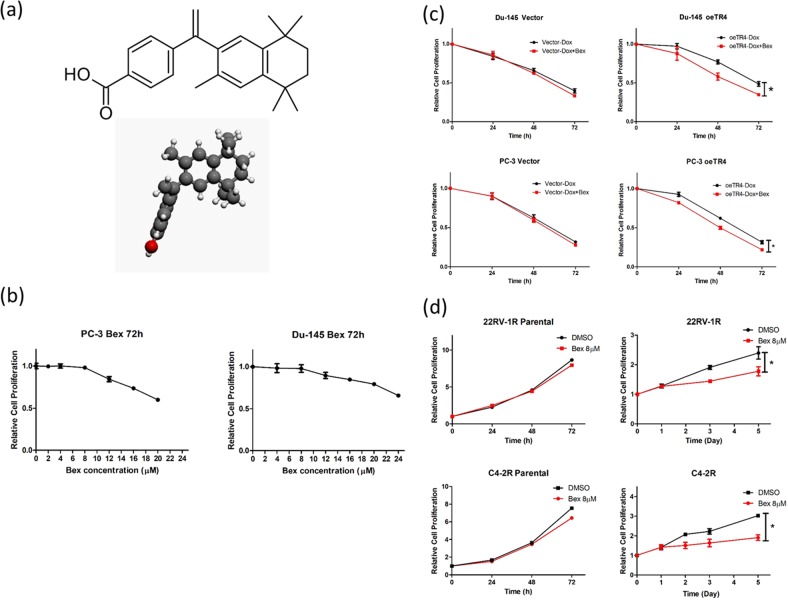
TR4 antagonist bexarotene (Bex) increased chemo-sensitivity of docetaxel in PCa cells: **a** Chemical structure of Bex, a retinoid derivative. **b** PC-3 and Du-145 cells were treated with different concentrations (0–20 μM for PC-3 and 0–24 μM for Du-145) of Bex within the clinical dosage range for 72 h and we found Bex concentrations below 8 μM have little influence on PC-3 and Du-145 proliferation, excluding the possibility that Bex may function via regulating its purported target, RAR/RXR. **c** Bex at 8 μM increased the chemo-sensitivity of docetaxel through TR4 in Du-145 and PC-3 cells treated with 2 nM DTX and oeTR4. **d** Parental 22RV-1 and C4-2 cells were treated with 8 μM Bex for 72 h and DTX-resistant 22RV-1R and C4-2R cells were treated with 8 μM Bex for 5 days. The 22RV-1R cells were maintained in 20 nM DTX and C4-2R dells maintained in 6 nM DTX. Cell proliferation results showed reduced cell proliferation in DTX-resistant cells, with no significant change in parental cells. Data are presented as mean ± SD, *p < 0.05

While targeting the TR4 with TR4-shRNAs led to increase the DTX sensitivity to better suppress the PCa cells (Fig. 2d, e), using shRNAs in the human clinical trials may still face many difficulties [23]. We are therefore interested to search for small molecules that can also effectively target TR4. TR4 is a member of nuclear receptor superfamily [24], and suitable antagonists should be able to suppress the TR4 function [25, 26]. Importantly, results from the analysis of the crystalline structure of the TR4 ligand-binding domain indicated that retinoid compounds including all *trans*-retinoic acid might be able to fit into the ligand-binding pocket to modulate the TR4 transactivation [26]. We therefore tested whether these retinoic acid derivatives might function as potential antagonists of TR4 to alter the DTX sensitivity to suppress the PCa cell growth. Among our initial screen of eight retinoid derivatives, we found bexarotene (Bex), (diagram in Fig. 3a), which was approved by the FDA for treating cutaneous T-cell lymphoma [27], was able to function as a TR4 antagonist to suppress TR4 function in PCa cells (see Fig. 3c, d). To exclude the possibility that Bex may function via regulating its purported target, RAR/RXR, we treated PC-3 and Du-145 cells with Bex alone for 72 h with different concentrations within the clinical dosage range and found Bex concentrations below 8 μM have little influence on PC-3 and Du-145 proliferation (Fig. 3b), suggesting that Bex at a low concentration in PCa cells is unlikely to function through RAR/RXR to alter the DTX sensitivity. However, when we compared the DTX sensitivity in PCa cells with different TR4 expression levels, we found that TR4 expression regulates the sensitivity of DTX, which can be modulated by the Bex treatment in both PC-3 and Du145 cells (Fig. 3c). Furthermore, since DTX-resistant PCa cells had elevated TR4 expression (See Fig. 2c), we treated parental and DTX-resistant 22RV-1 and C4-2 cells with Bex, and found that 8 μM Bex could reduce cell proliferation in DTX-resistant cells, but had no significant change in parental cells (Fig. 3d).

Together, these results (Fig. 3a–d) suggest that Bex may increase DTX sensitivity via repressing the TR4 activity in the PCa cells.

### Bex can function as TR4 antagonist to regulating TR4 transactivation

**Fig. 4:**
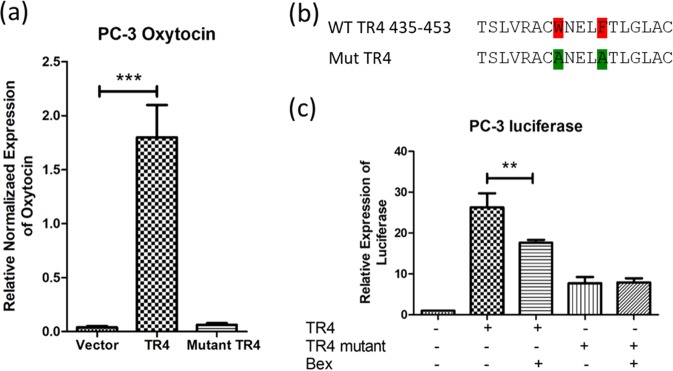
Bex decreased reporter activity of oxytocin promoter in PC-3 cells: **a** One kilobase promoter region of oxytocin was constructed into pGL3-basic vector, and transfected into PC-3 cells. The reporter activity was significantly increased in response to wild-type TR4 but not mutant TR4 expression. **b** Wild type (WT) and Mutation (Mut) of the ligand-binding domain of TR4. **c** A total of 1 × 10^3^ cells/well were plated in 24-well plates and cDNA were transfected using Lipofectamine. PC-3 cells were transfected with TR4 expression construct (pcDNA3-TR4) or pcDNA3 as control together with pGL3-oxytocin and pRL-TK for 24 h. Then, PC-3 cells were treated with 6 μM Bex or DMSO for 24 h. Luciferase activity was measured by the Dual-Luciferase Assay. The results showed 6 μM Bex could suppress luciferase reporter activity in the WT TR4 but not Mut TR4 group. Data are presented as mean ± SD, **p < 0.01, ***p < 0.001

To further confirm Bex can alter the TR4 transactivation, we established a reporter assay in PC-3 cells with the promoter of TR4 target gene, oxytocin (Fig. 4a). The results from the luciferase reporter assay via transient transfection of both wild type (WT) and mutant (Mut) TR4 (Fig. 4b) revealed that 6 μM Bex could suppress the TR4 transactivation with WT TR4 and had little effect with the Mut TR4 (Fig. 4c). These results confirm the above results and prove that Bex can function as a TR4 antagonist to regulate TR4 transactivation.

### Bex decreases TR4 transactivation via altering the transcription from a TR4 target gene natural promoter

**Fig. 5:**
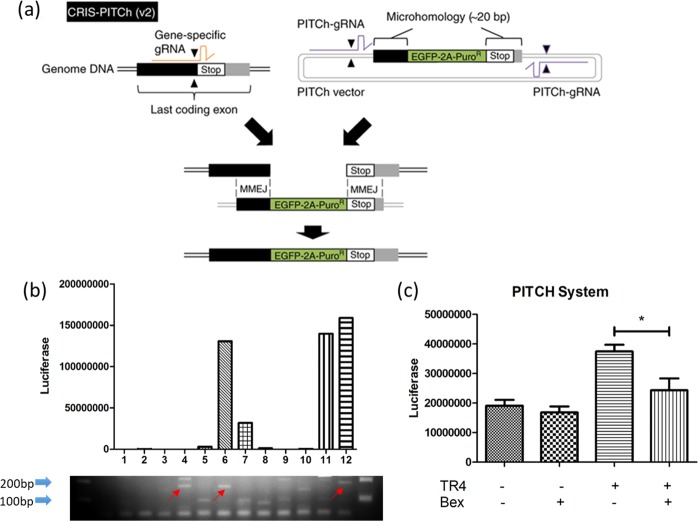
Bexarotene decreased TR4’s transactivation via regulating the transcription from a natural promoter: **a** Diagram of the PITCh system with CRISPR-CAS9 technique to establish stable cell lines with luciferase inserted at the genomic location in the oxytocin gene in 293T cells. **b** Twelve single clones were expanded upon selection with 2 μM puromycin, six of which had luciferase value increases in response to oeTR4 through lentiviral transfection, and three clones (#6, #11, and #12) had the correct genomic structure indicating that luciferase construct was indeed successfully inserted at the intended genomic location through genomic PCR assays (lower panel). **c** Selected cloned cells, 1 × 10^3^ of cells/well, were plated in 24-well plates and transfected with vector or oeTR4 for 24 h. After transfection, cells were treated with DMSO or 8 μM Bex for 24 h. Renilla luciferase ORF was measured by the Dual-Luciferase Assay. The results showed TR4 could significantly increase the luciferase activity, which can be reduced with Bex treatment in the PITCh system. In **b** and **c**, data are presented as mean ± SD, *p < 0.05

To test whether Bex can influence the ability of TR4 to regulate its target gene in its natural chromatin environment, we used the PITCh system with CRISPR-CAS9 technique to establish stable cell lines with luciferase inserted at the genomic location in the oxytocin gene of 293T cells (Fig. 5a). Then, 12 single clones were expanded upon selection with 2 μM puromycin, six of which had luciferase value increases in response to overexpression of TR4 through lentiviral expression. And three clones (6, 11, and 12) had the correct genomic structure indicating that luciferase construct was indeed successfully inserted at the intended genomic location verified by genomic PCR assays (Fig. 5b). We then selected two clones for further analysis, and results indicated that TR4 could significantly increase the luciferase activity which could be reduced with 8 μM Bex treatment, suggesting that TR4 can regulate its target gene in its natural environment which can also be suppressed by Bex (Fig. 5c, 1 clone data shown).

### Mechanism dissection of how TR4 can alter the chemo-sensitivity in PCa cells: via altering the lincRNA-p21 expression

**Fig. 6:**
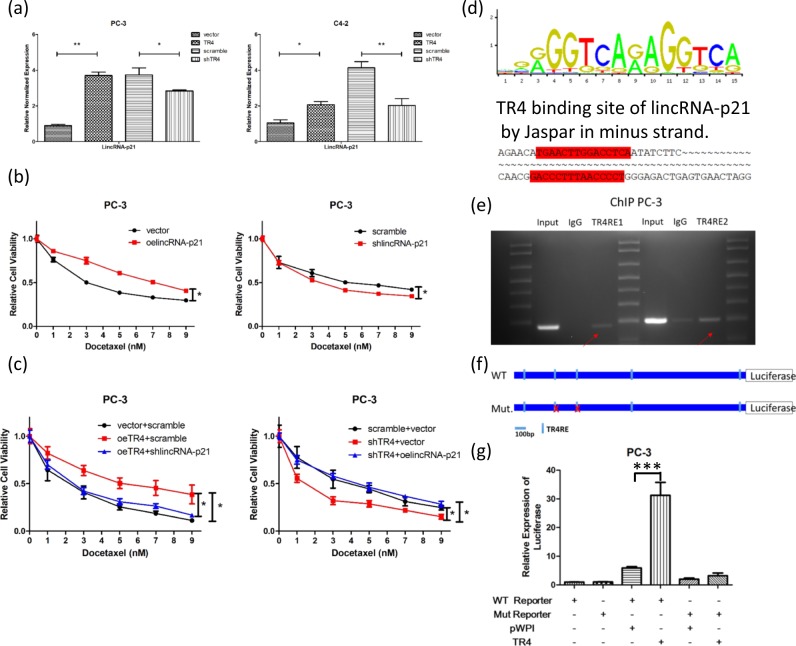
TR4 regulated chemo-sensitivity in PC-3 and C4-2 cells through lincRNA-p21: **a** Overexpressing TR4 (oeTR4) increases lincRNA-p21 level while knocking down TR4 (shTR4) decreases it in PC-3 and C4-2 cells. **b** PC-3 cells were transfected with vector/oeLincRNA-p21, scramble/shLincRNA-p21, cultured in 0–9 nM DTX concentrations for 48 h. Cell viability results showed that oeLincRNA-p21 could increase DTX resistance and shLincRNA-p21 decreases DTX resistance. **c** PC-3 cells were first transfected with vector/oeTR4 or scramble/shTR4 for 24 h. After successfully transfection, cells with oeTR4 were transfected with scramble/shLincRNA-p21 and cells with shTR4 were transfected with vector/oeLincRNA-p21. After double transfection, PC-3 cells were treated with 0–9 nM DTX concentrations for 48 h. The results showed shLincRNAs-p21 could reverse oeTR4-related DTX resistance and oeLincRNA-p21 could reverse shTR4-related DTX-sensitization. **d** Predicted TR4 binding site on lincRNA-p21 by Jaspar in minus strand. **e** The ChIP assay showed that TR4 can transcriptionally regulate lincRNA-p21 expression through the TR4RE binding sites upstream of the lincRNA-p21 promoter. **f** Luciferase reporter constructed with 3 kb upstream sequence containing WT or Mut lincRNA-p21. **g** Adding TR4 significantly increased the reporter activity in PC-3 cells transfected with the WT construct but not the Mut construct. In **a**–**c** and **g**, the data are presented as mean ± SD, *p < 0.05, **p < 0.01, ***p < 0.001

To further dissect the molecular mechanism of how TR4 regulates the chemo-sensitivity in the PCa cells, we examined the known target genes of chemotherapy, and found that lincRNA-p21 is induced in response to TR4 in PCa cells (Fig. 6a). Indeed, we also found that lincRNA-p21 is functionally important for the PCa chemo-sensitivity. The cell viability results showed that overexpressing lincRNA-p21 increased DTX resistance and knocking down lincRNA-p21 decreased DTX resistance (Fig. 6b) in PC-3 cells. Importantly, results from an interruption approach via silencing lincRNA-p21 expression could reverse/block TR4-induced DTX resistance while overexpressing lincRNA-p21 could block shTR4-induced DTX sensitivity in PC-3 cells (Fig. 6c).

Mechanism study further revealed that TR4 could regulate lincRNA-p21 expression via direct binding to the potential TR4REs upstream of lincRNA-p21, based on bioinformatics prediction as well as Chromatin Immunoprecipitation (ChIP) in vivo binding analysis (Fig. 6d, e). This conclusion was further confirmed by the results from promoter luciferase assays with WT (and not Mut) lincRNA-p21 promoter in response to TR4 overexpression (Fig. 6f, g).

Together, the results from Fig. 6a–g suggest that TR4 can alter the chemo-sensitivity via direct binding to TR4REs located on the lincRNA-p21 upstream region to alter the lincRNA-p21 expression.

### Mechanism dissection of how TR4/lincRNA-p21 can alter the chemo-sensitivity in PCa cells: via altering the HIF-1α/VEGF-A signaling

**Fig. 7:**
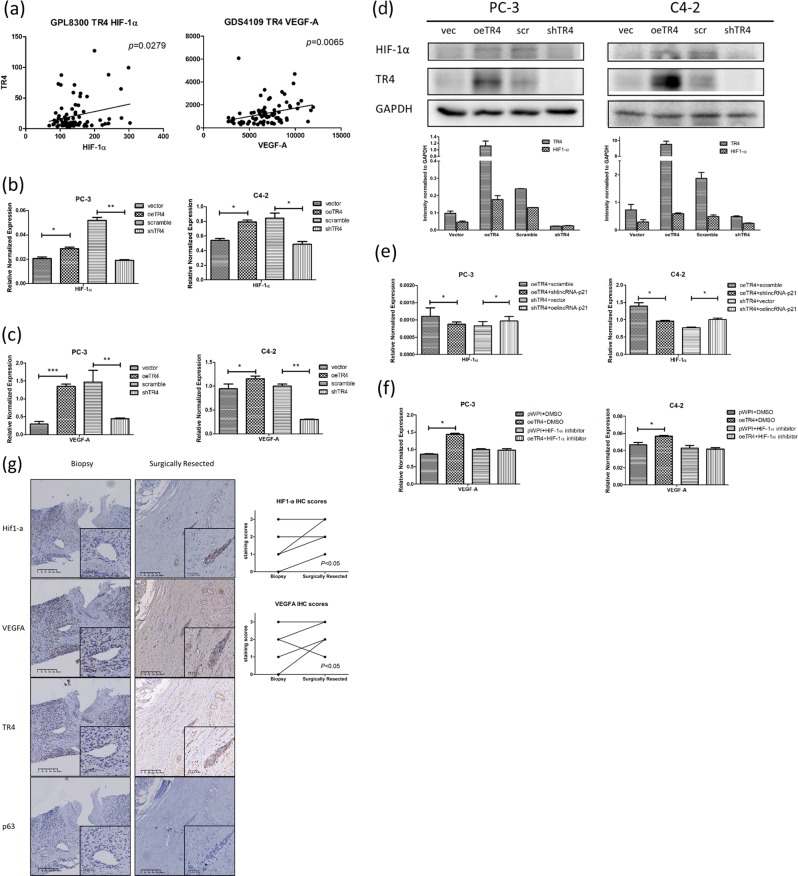
Regulation of the TR4/lincRNA-p21/HIF-1α/VEGF-A pathway: **a** Expression levels of TR4 are positively correlated with HIF-1α (left) and VEGF-A (right) in PCa tissues from analysis of Datasets GPL8300 and GDS4109. **b**, **c** HIF-1α and VEGF-A increased when TR4 was overexpressed but decreased when TR4 was knocked down in PC-3 and C4-2 cells. **d** Western blot (upper) and quantification (lower) showed HIF-1α increased when TR4 was overexpressed, but decreased when TR4 was knocked down in PC-3 and C4-2 cells. **e** Silencing lincRNAs-p21 could reverse oeTR4-related HIF-1α increase and overexpressing lincRNA-p21 could reverse shTR4-related HIF-1α decrease. **f** Treatment with a HIF-1α inhibitor at 40 μM (Tocris Bioscience, MO, USA) could reverse oeTR4-related VEGF-A increase in PC-3 and C4-2 cells. **g** Serial section staining of HIF-1α, VEGF-A, TR4, and P63 in clinical tissues (left panels), by comparing biopsy samples at first diagnosis and surgically resected samples after DTX treatment at ×100 and ×400 magnification. IHC score comparisons (right panels) between biopsy samples and surgically resected samples in the same patients after DTX treatment. Due to the limited size of biopsy samples, only eight cases were included in this assay (details in Supplementary data Table S2) and significant differences were found in HIF-1α and VEGF-A stains (*p* < 0.05). Quantitative scoring methods were applied as in Fig. 1, see Legend. In **b–f**, the data are presented as mean ± SD, *p < 0.05, **p < 0.01, ***p < 0.001

Finally, to dissect the mechanism how TR4/lincRNA-p21 axis can alter the chemo-sensitivity in PCa, we focused on the HIF-1α, as an early study indicated that lincRNA-p21 might be linked with HIF-1α in regulating Warburg effect in cancer [28]. We first applied the database analysis from GPL8300 and GDS4109, and found the positive correlation among TR4, HIF-1α, and VEGF-A (Fig. 7a). Moreover, the expression of HIF-1α and its target gene, VEGF-A, were altered in response to TR4 expression in both PC-3 and C4-2 cells (Fig. 7b–d). In addition, this functional connection between TR4 and HIF-1α appeared to be through lincRNA-p21 as silencing lincRNA-p21 could reverse oeTR4-related HIF-1α increase while overexpressing lincRNA-p21 could reverse shTR4-related HIF-1α decrease (Fig. 7e) in both cell lines. This newly identified TR4/lincRNA-p21/HIF-1α/VEGF-A signaling was also validated through the treatment with the HIF-1α inhibitor as it could reverse oeTR4-related VEGF-A increase (Fig. 7f). Furthermore, we used serial section staining of HIF-1α, VEGF-A, TR4, and P63 in clinical tissues (Fig. 7g), by comparing biopsy samples at first diagnosis and surgically resected samples after DTX treatment (details in Supplementary data Table S2). Significant upregulations of HIF-1α and VEGF-A were found in PCa patients after DTX treatment, *p* < 0.05.

Together, the results from Fig. 7a–g suggest that TR4 can alter the DTX sensitivity via altering the TR4/lincRNA-p21/HIF-1α/VEGF-A signaling in the PCa cells.

## Discussion

DTX is likely to remain a mainstay of treatment for men with mCRPC for the foreseeable future. Similar to most chemotherapeutic agents, the impact of this chemotherapy is limited due to clinical resistance. A significant proportion of patients do not respond to DTX chemotherapy, despite the overall survival benefit seen in the entire population of patients with mCRPC [4]. In addition, responses to DTX are not durable; progression-free survival on DTX treatment approaches 0% by 3 years [6]. DTX exerts its cytotoxic activity through the stabilization of microtubules [7], which are filamentous polymers composed of α- and β-tubulin heterodimers, that may play critical roles for cell division and are thought to be the primary target of DTX action [4].

Our clinical sample survey from a cohort of 14 metastatic PCa patients before and after DTX chemotherapy indicated that TR4 expression was correlated with DTX resistance. This significant finding suggests that TR4 may not only serve as a potential biomarker for the efficacy of DTX chemotherapy, but more importantly TR4 could be a target of adjuvant therapy through a small molecule, such as Bex. Indeed, our results from multiple PCa cell lines support the role of TR4 in regulating DTX sensitivity, and adding Bex can enhance the DTX sensitivity, thus could be potentially used in those patients either not responsive or resistant to DTX. It is possible that other small compounds, such as metformin and polyunsaturated fatty acids that have been reported earlier to regulate TR4 function, might also be able to serve as adjuvant therapy to enhance DTX sensitivity [20]. However, this potential repurposing of existing drugs, although promising, requires further studies to identify the potential side-effects and tolerable dosages.

Mechanistically we found that lincRNA-p21 might mediate the effect of TR4 in enhancing DTX sensitivity. LincRNA-p21 is a transcriptional target of p53 and has been proposed to act in translation via several mechanisms including repressing genes in the p53 transcriptional network and regulating mRNA translation, protein stability, and apoptosis [16, 29, 30]. In the vascular system, lincRNA-p21 regulated neointima formation, vascular smooth muscle cell proliferation, apoptosis, and atherosclerosis by enhancing p53 activity [31]. LincRNA-p21 also has been identified as a regulator for the Warburg effect and stem cell pluripotency [28, 31]. In hypoxic hepatocellular and glioma tumor cells, knocking down lincRNA-p21 could enhance radio-sensitivity through HIF-1/Akt/mTOR/P70S6K signaling [32], while in human colorectal cancer, lincRNA-p21 enhanced the sensitivity of radiotherapy by targeting the Wnt/β-catenin signaling [33]. In head and neck cancer, Fayda et al. found pretreatment or posttreatment circulating lincRNA-p21 levels were not informative for radical chemoradiotherapy treatment response [30]. In PCa cells (Du-145 and LNCaP), lincRNA-p21 suppressed cell development through inhibition of PKM2 [19], while clinical samples from patients with PCa had significant higher exosomal lincRNA-p21 levels than patients with BPH [17]. Here we found that TR4 increased chemo-resistance through lincRNA-p21 and transcriptionally regulated lincRNA-p21 expression likely through the TR4RE binding sites upstream of the lincRNA-p21 promoter, indicative of regulation in a p53-independent pathway. Our studies thus suggested the possibility that TR4 and in extension, small molecule regulators of TR4, might also be therapeutically relevant in those processes involving lincRNA-p21 through transcriptional regulation of its expression. Further studies are required to test these hypotheses.

In summary we identified a novel role of TR4 that may function via altering the TR4/lincRNA-p21/HIF-1α/VEGF-A signaling to modulate the DTX resistance in PCa, and targeting this newly identified signaling with Bex, an FDA-approved drug, may lead to increase DTX chemo-sensitivity to better suppress the mCRPC progression.

## Materials and methods

### Clinical tissues

Clinical samples of PCa tissues collected for research were obtained from the patients hospitalized between January 1, 2007 and December 31, 2016 in the Department of Urology, Zhejiang Provincial People’s Hospital, Hangzhou, China. Patients were informed of the scientific ethics and signed Consent Forms for their tissues to be used only for research studies. The study was approved by the Ethic Committee of Zhejiang Provincial People’s Hospital (Hangzhou, China).

### Reagents and materials

TR4 and HIF-1α antibodies were purchased from ABCam (MA, USA). HIF-1α rabbit antibody (ab51608, Abcam) was used for IHC, anti-TR4 rabbit antibody (ab109301, Abcam) for WB and ChIP, and anti-TR4 mouse antibody (ab58365, Abcam) for IHC of best nuclear staining. VEGFA (PB0084) and P63 (PB0164) were from Boster Bio (CA, USA). GAPDH, beta-ACTIN antibodies, and normal rabbit IgG were from Santa Cruz Biotechnology (CA, USA). Anti-mouse/rabbit antibody was from Invitrogen (NY, USA). DTX and Bex were purchased from Sigma-Aldrich (MO, USA) and HIF-1α inhibitor was purchased from Tocris Bioscience (MO, USA).

### Cell culture

We used human PCa cell lines C4-2, 22RV-1, PC-3, DU145, and human benign prostate cell lines WPMY-1 (immortalized human prostatic stromal cell) and BPH-1 (immortalized human prostatic epithelial cell), which were obtained from American Type Culture Collection (ATCC) and maintained in RPMI-1640 media (Invitrogen). DTX-resistant C4-2 and CWR22Rv-1 (22RV-1) cells were obtained as previous described [34], named as C4-2R and 22RV-1R, and maintained in RPMI-1640 media with DTX concentration of 6 and 20 nM, respectively. 293T cells (from ATCC) were maintained in DMEM media (Invitrogen). All media contained 1% penicillin and streptomycin, supplemented with 1% l-glutamate and 10% fetal bovine serum. All cells were cultured in a 5% (v/v) CO_2_ humidified incubator at 37 °C.

### Lentivirus packaging

293T cells were co-transfected with pLKO.1/pLKO.1-TR4/pLKO.1-lincRNA-p21 or pWPI/pWPI-TR4/pWPI-lincRNA-p21, together with pMD2G envelope plasmid and psAX2 packaging plasmid. Standard calcium phosphate transfection method was used and after incubating for 48 or 72 h, the lentivirus soup was collected for immediate use and/or frozen at −80 °C for later use. The PCa cells were transfected under LipofectAMINE 3000 (Invitrogen) reverse transfection protocol, according to the manufacturer’s instructions.

### Cell counting assay

Cells were seeded on 96-well plates at a density of 1 × 10^3^ per well. After treatment, plates were collected at selected times, emptied by overturning on absorbent toweling and stored frozen at −80 °C. On the day of assay, 100 μl distilled water was added to the wells, the plates incubated for 1 h at room temperature, frozen at −80 °C for 2 h, and then thawed until reaching room temperature. Then, 100 μl of the fluorochrome at 20 pg/ml in TNE buffer (10 mM Tris, 1 mM EDTA, 2 M NaCl, pH 7.4) was added to each well. This yielded a final concentration of 10 pg/ml of Hoechst 33258 (Thermo Fisher Scientific, IL, USA) per well in 200 μl with a final concentration of 1 M NaCl. Plates were wrapped in foil and shaked on an orbital shaker for 15 min. Then, 100 μl from the 200 μl mixture from each well was transferred to 96-well black microplates (Corning, NY, USA) for measuring the fluorescence (plate reader excitation at 361 nm and emission measured at 486 nm wavelength). Several experiments were performed with triplicate data points and mean values ± SD were presented.

### Quantitative real-time PCR

Total RNAs were isolated using Trizol reagent (Invitrogen) and 1–2 μg of total RNA was subjected to reverse transcription using Superscript III transcriptase (Invitrogen). Quantitative real-time PCR was conducted using a Bio-Rad CFX96 system with SYBR green to determine the mRNA expression level of a gene of interest. Expression levels were normalized to the expression of GAPDH mRNA. The primers for TR4 are: Forward, 5′-TCC CCA CGC ATC CAG ATA ATC-3′; Reverse, 5′-GAT GTG AAA ACA CTC AAT GGG C-3′. GAPDH: Forward, 5′-GGA GCG AGA TCC CTC CAA AAT-3′; Reverse, 5′-GGC TGT TGT CAT ACT TCT CAT GG-3′. HIF-1α: Forward, 5′-GTG TAC CCT AAC TAG CCG AGG-3′; Reverse, 5′-GCA GTG CAA TAC CTT CCA TGT T-3′. VEGF-A: Forward, 5′-CTC AGA TGT GAC AAG CCG AG-3′; Reverse, 5′-CCA GAC CCA AGA CAA ATG CC-3′. lincRNA-p21: Forward: 5′-ATA ACC CGA GCT GAA GGA GG-3′; Reverse, 5′-CTC TTG GTG GAG GCA GAG AA-3′.

### Western blot analysis

Cells were lysed in RIPA buffer and proteins (20–50 μg) were separated on 8–10% SDS/PAGE gel, and then transferred onto PVDF membranes (Millipore, MA, USA). Membranes were blocked with nonfat milk at room temperature for 1 h and incubated overnight at 4 °C with proper dilutions of primary antibodies against GAPDH, beta-ACTIN, TR4 (ab109301, Abcam), HIF-1α (ab51608, Abcam). The next day after three 10 min rinses with TBST, horseradish peroxidase (HRP)-conjugated secondary antibody was added for incubation for 1 h at the concentration of 1:5000 at room temperature, and then rinsed three times for 10 min with TBST. The bands were visualized using ECL system (Thermo Fisher Scientific, NY, USA). Protein bands were visualized on chemiluminescence system (Bio-Rad, CA, USA).

### Luciferase reporter gene assays

The 1 kb promoter region of oxytocin was constructed into pGL3-basic vector (Promega, WI, USA). Then 1 × 10^3^ of cells/well were plated in 24-well plates and cDNA were transfected using Lipofectamine (Invitrogen) according to the manufacturer’s instructions. PC-3 cells were transfected with TR4 expression construct (pcDNA3-TR4) or pcDNA3 as control together with pGL3-oxytocin and pRL-TK for 24 h. Three hundred and fifty base pair fragments of TR4 3′UTR containing WT or Mut responsive elements were cloned into the psiCHECK™-2 vector construct (Promega) downstream of the Renilla luciferase ORF. PC-3 cells were treated with 6 μM Bex or DMSO for 24 h. Luciferase activity was measured by the Dual-Luciferase Assay (Promega) according to the manufacturer’s manual.

### PITCh system establishment

We followed the recently published PITCh system [35] to construct a reporter cell line that can faithfully reflect the TR4 activation status at the genome level. We found that among many TR4 target genes, oxytocin appears to be readily responsive to the TR4 expression in PC-3 cells. And by utilizing the CRISPR-CAS9-assisted genome engineering technique, we inserted a luciferase gene at the oxytocin gene locus in the genome and selected stably transduced cells with 1.5 μg/ml puromycin.

### Chromatin Immunoprecipitation Assay (ChIP)

Cells were cross-linked with 4% formaldehyde for 10 min, collected, and sonicated with a predetermined power to yield genomic DNA fragments of 300–1000 bp long. Cell lysates were precleared sequentially with normal rabbit IgG (sc-2027, Santa Cruz Biotechnology) and protein A-agarose. Then, 2.0 µg anti-TR4 rabbit antibody (ab109301, Abcam) was added into the cell lysates and incubated overnight at 4 °C. IgG was added for the negative control. Specific primer sets were designed to amplify a target sequence within the human lincRNA-p21’s promoter. PCR products were identified by agarose gel electrophoresis.

### H&E and immunohistochemical (IHC) staining

Tissues were fixed in 10% (v/v) formaldehyde, embedded in paraffin, and then 4 μm sections were cut and used for H&E staining with primary antibodies. The slides were treated with EDTA for antigen retrieval and incubated with endogenous peroxidase blocking solution to inhibit endogenous peroxidase. HRP-streptavidin conjugated secondary antibody (Vector Laboratories) was used and detected by the DAB kit (Vector Laboratories). Primary antibodies were visualized by slides scanner (KF-PRO-005, Ningbo, China). We used quantitative scoring methods as scores 0, 1+, 2+, and 3+, representing positive cells of none or rare, <25%, 25–75%, and >75%, respectively. According to the product datasheet, TR4 antibody (ab58365, Abcam) stains nuclei. HIF1a (ab51608, Abcam) antibody stains cytoplasm and nuclei (mainly), VEGFA antibody (PB0084, Boster) stains cytoplasm, and P63 antibody (PB0164, Boster) that is used as a quality control stains nuclei.

### Tumor sphere formation

After microscopic examination for single-cell suspension, cells were plated in ultra-low adhesion 48-well plates at a density of 1 × 10^3^ cells/well and grown in serum-free RPMI-1640 and matrigel (BD Biosciences, 1:1) media containing 20 ng/ml of epidermal growth factor (EGF, R&D Systems, Minneapolis, MN, USA). After 7 days in culture, the number of spheres was quantified by counting spheres in three random fields (×40) under a phase contrast microscope (Olympus, Japan).

### Statistics

Data are expressed as mean ± SD from at least three independent experiments with data points performed in triplicate. Statistical analyses involved paired *t*-test, One-way ANOVA and Log-rank (Mantel-Cox) with SPSS 17.0 (SPSS Inc., Chicago, IL) or GraphPad Prism 6 (GraphPad Software, Inc., CA, USA). *p* < 0.05 was considered statistically significant.

## Supplementary information

Supplementary data table S1

Supplementary data table S2
