# Supplementary material for: Targeting TR4 nuclear receptor with antagonist bexarotene increases docetaxel sensitivity to better suppress the metastatic castration-resistant prostate cancer progression
Source: Oncogene. 2019 Nov 20;39(9):1891–903. doi: 10.1038/s41388-019-1070-5 (PMC7044111; doi:10.1038/s41388-019-1070-5)
Supplement: Supplementary file 1 — Supplementary data table S1 [file 41388_2019_1070_MOESM1_ESM.docx]

**Table S1.** TR4 expressions after docetaxel treatment in 14 PCa patients

|  |  |  |  |  | **IHC Scores** | |  |
| --- | --- | --- | --- | --- | --- | --- | --- |
| **Case Num.** | **Age** | **DTX treatment (Month)** | **Gleason Score at diagnosis** | **Metastasis** | **pre-DTX  treatment** | **post-DTX treatment** | **Surgical Intervetion  after Chemotherapy** |
| 1 | 68 | 3 | 4+5 | bone | + | ++ | TURP |
| 2 | 72 | 8 | 4+4 | bone and liver | + | +++ | TURP |
| 3 | 72 | 1 | 4+4 | bone (oligo) | + | ++ | Prostatectomy |
| 4 | 73 | 6 | 5+4 | bone | ++ | +++ | TURP |
| 5 | 67 | 6 | 4+5 | bone and liver | ++ | +++ | TURP |
| 6 | 72 | 7 | 4+5 | bone | + | ++ | TURP |
| 7 | 66 | 4 | 4+3 | bone | + | ++ | TURP |
| 8 | 65 | 5 | 3+4 | bone | ++ | ++ | TURP |
| 9 | 78 | 4 | 4+4 | bone | + | +++ | TURP |
| 10 | 84 | 5 | 4+5 | bone | + | ++ | TURP |
| 11 | 78 | 6 | 4+4 | bone | - | ++ | TURP |
| 12 | 71 | 1 | 4+4 | bone (oligo) | + | ++ | prostatectomy |
| 13 | 78 | 6 | 4+5 | bone | ++ | ++ | TURP |
| 14 | 76 | 5 | 5+4 | bone | +++ | +++ | TURP |

TR4 expression increased after docetaxel treatment in 14 PCa patients. In the same patients, comparing the TR4 expressions of biopsy samples with surgically resected samples after DTX treatment. 11 cases had TR4 levels increase after DTX treatment while 3 cases had no change between before and after DTX treatment.
