# Supplementary material for: Targeting TR4 nuclear receptor with antagonist bexarotene increases docetaxel sensitivity to better suppress the metastatic castration-resistant prostate cancer progression
Source: Oncogene. 2019 Nov 20;39(9):1891–903. doi: 10.1038/s41388-019-1070-5 (PMC7044111; doi:10.1038/s41388-019-1070-5)
Supplement: Supplementary file 2 — Supplementary data table S2 [file 41388_2019_1070_MOESM2_ESM.docx]

**Table S2.** HIF1-α and VEGF-A expressions after docetaxel treatment in 8 PCa patients

|  | **HIF1-αIHC Scores** | |  | **VEGF-A IHC Scores** | |
| --- | --- | --- | --- | --- | --- |
| **Case Num.** | **pre-DTX  treatment** | **post-DTX treatment** |  | **pre-DTX  treatment** | **post-DTX treatment** |
| 4 | + | +++ |  | +++ | +++ |
| 5 | ++ | ++ |  | ++ | +++ |
| 7 | + | +++ |  | ++ | +++ |
| 9 | + | ++ |  | + | ++ |
| 10 | ++ | ++ |  | ++ | + |
| 11 | - | ++ |  | - | ++ |
| 12 | ++ | ++ |  | + | ++ |
| 14 | +++ | +++ |  | ++ | +++ |

IHC score comparisons between biopsy samples and surgically resected samples in the same patients after DTX treatment. Due to the limited size of biopsy samples, only 8 cases were included in this assay. Significant differences were found in both groups (p<0.05).
